# The Current State of Nanoparticle-Induced Macrophage Polarization and Reprogramming Research

**DOI:** 10.3390/ijms18020336

**Published:** 2017-02-06

**Authors:** Xiaoyuan Miao, Xiangfeng Leng, Qiu Zhang

**Affiliations:** 1 School of Chemistry and Chemical Engineering, Shandong University, Jinan 250100, China; miaoxiaoyuan91@163.com; 2 Department of Plastic Surgery, The Affiliated Hospital of Qingdao University, Qingdao 266003, China

**Keywords:** nanoparticles, macrophage, polarization, reprogramming

## Abstract

Macrophages are vital regulators of the host defense in organisms. In response to different local microenvironments, resting macrophages (M0) can be polarized into different phenotypes, pro-inflammatory (M1) or anti-inflammatory (M2), and perform different roles in different physiological or pathological conditions. Polarized macrophages can also be further reprogrammed by reversing their phenotype according to the changed milieu. Macrophage polarization and reprogramming play essential roles in maintaining the steady state of the immune system and are involved in the processes of many diseases. As foreign substances, nanoparticles (NPs) mainly target macrophages after entering the body. NPs can perturb the polarization and reprogramming of macrophages, affect their immunological function and, therefore, affect the pathological process of disease. Optimally-designed NPs for the modulation of macrophage polarization and reprogramming might provide new solutions for treating diseases. Systematically investigating how NPs affect macrophage polarization is crucial for understanding the regulatory effects of NPs on immune cells in vivo. In this review, macrophage polarization by NPs is summarized and discussed.

## 1. Introduction

With unique physicochemical characteristics, nanoparticles (NPs) are widely used in the biomedical field for disease diagnosis and therapy as antigen carriers [1], imaging labels [2], and therapeutic drugs [3,4,5]. In addition, the wide application of NPs in cosmetics [6], food [7], and medical products [8] has increased their entry into the environment.

The wide use of NPs increases the chance for human exposure [4]. NPs can be absorbed into the circulatory system and redistributed into various organs after environmental exposure [4]. Meanwhile, the safety of NPs in vivo has caused much concern. Emerging evidence has shown that NPs can disturb the immune system [5] and affect the process of diseases such as inflammation [9,10], allergies [11], and tumors [12] in animal models.

As one of the most effective cell types in the emergency response system of the human body, macrophages modulate host defense, inflammatory processes, and homeostasis [13]. With powerful abilities of cell engulfment, antigen presentation and cytokine secretion, macrophages govern the initiation and resolution stages of innate and adaptive immunity [14].

Macrophage polarization and reprogramming are crucial for their function. Responding to different microenvironment, primary macrophages (M0) can be polarized toward pro-inflammatory (M1) or anti-inflammatory (M2) phenotypes [15]. Furthermore, the polarized macrophage can also be reprogrammed by reversing its phenotype [15]. Various polarized macrophages play different roles in immune regulation, inflammation, tissue remodeling, proliferation, and metabolism. For example, pro-inflammatory M1 macrophages are key effector cells in the resistance to intracellular pathogens and tumor growth [12,16], while anti-inflammatory M2 macrophages are associated with immunosuppression, promotion of tissue remodeling and tumor progression [17]. Macrophage polarization and reprogramming play important roles in the maintenance of immune system homeostasis [13] and provide solutions for treating associated diseases [18].

NPs are foreign substances, and macrophages play crucial roles in the recognition, processing, and clearance of NPs in vivo [19]. Different macrophage phenotypes (M1 or M2) have distinct uptake capacity for NPs [20]. Meanwhile, NPs can prime the macrophage toward various polarization states as a stimulus in the microenvironment [21,22,23]. Different NPs can differentially modulate macrophage polarization and reprogramming [22,23,24]. Interactions of NPs with macrophages have attracted a great deal of attention in medical applications [25] and toxicological studies. A more in-depth understanding of the effects of NPs on macrophage polarization is crucial for us to modulate the biological effects of NPs in vivo and design nanotechnology-based therapies [26,27].

## 2. Macrophage Polarization

Macrophages are plastic and heterogeneous immune cells [28]. Resting macrophages undergo various functional changes in order to adjust to the changes in the residing milieu. This dynamic process of macrophage functional change is defined as macrophage polarization [18]. The primary phenotype of a macrophage without stimulation or in the resting cells is referred to as an M0 macrophage. The M1 and M2 phenotypes are the two main activated states of macrophages [29]. M0 macrophages can be induced by environmental signals toward M1 or M2 polarization [15].

The phenotype of the polarized macrophage is tightly linked to the microenvironment within which they reside. Many local environmental stimuli can modulate macrophage activating states [30]. For example, in response to lipopolysaccharides (LPS) or interferon-γ (IFN-γ) stimulus, macrophages can be induced into the M1 phenotype [31], also called “classically activated macrophages”. Alternatively, in response to interleukin-4 (IL-4)/interleukin-13 (IL-13) or interleukin-10 (IL-10), M0 macrophages can be induced into the M2 phenotype, commonly called “alternatively activated macrophages”. Further studies demonstrated that M2-polarized macrophages can be further divided into three subpopulations, M2a, M2b, and M2c, according to whether they are stimulated by IL-4, IL-13, or IL-10 [32]. Among them, M2a, commonly called M2, has been more extensively studied. In addition, M1 activated macrophages could be re-educated into the M2 phenotype, and vice versa, by further changes in the stimuli. This process is referred to as macrophage reprogramming or repolarization [33,34].

Different phenotypes of macrophages play specific roles in the progression of correlative diseases [35]. For example, in inflammation, the M1 macrophage is predominantly responsible for the clearance of intracellular pathogens by releasing pro-inflammatory cytokines to recruit and activate T cells and B cells during the early phase of inflammation. However, during the resolution of inflammation, macrophages switch to the anti-inflammatory M2-like phenotype [35]. Anti-inflammatory M2 macrophages are associated with the promotion of tissue remodeling.

The optimal control of macrophage polarization and reprogramming could provide a novel approach to treat related diseases. For example, macrophage reprogramming from the M1 phenotype to the M2 phenotype can be used to treat chronic inflammatory diseases [36]. The phenotypic shift of M2 macrophages toward an M1 macrophage subtype is beneficial for the early phases of inflammation and cancer immunotherapy [37].

### 2.1. Characterization Polarized Macrophage Phenotype

Cytokines are vital biomarkers of the activated state of macrophages. The primary markers of each distinct phenotype are different. The M1 phenotype produces high amounts of pro-inflammatory Th1-type cytokines, such as interleukin-12 (IL-12), interleukin-1 β (IL-1β), TNF-α, interleukin-18 (IL-18), interleukin-6 (IL-6) [38], and low amounts of interleukin-10 (IL-10). However, the M2 phenotype is characterized by high levels of the anti-inflammatory cytokine IL-10 and low levels of IL-12 and interleukin-23 (IL-23).

In addition to cytokines, membrane receptors, chemokines, and enzymes are indispensable markers to characterize the polarized state of macrophages. The M1 phenotype highly expresses membrane receptors for cluster of differentiation 86 (CD86) and toll-like receptor 4 (TLR4), but the M2 phenotype exhibits high levels of cluster of differentiation 163 (CD163) and cluster of differentiation 206 (CD206). Chemokine (C-C motif) ligand 5 (CCL5) and chemokine (C-C motif) ligand 2 (CCL2) are the chemokines associated with the M1 phenotype, while chemokine (C-C motif) ligand 17 (CCL17) and chemokine (C-C motif) ligand 22 (CCL22) are the chemokines associated with the M2 phenotype. Enzymes, inducible nitric oxide synthase (iNOS) and arginase-1 (Arg-1), are, respectively, expressed in the M1 and M2 phenotype. Arg-1 and chitinase 3-like 3 (Ym-1) are M2 markers for murine macrophages [39].

### 2.2. Mechanisms of Macrophage Polarization

**Figure 1:**
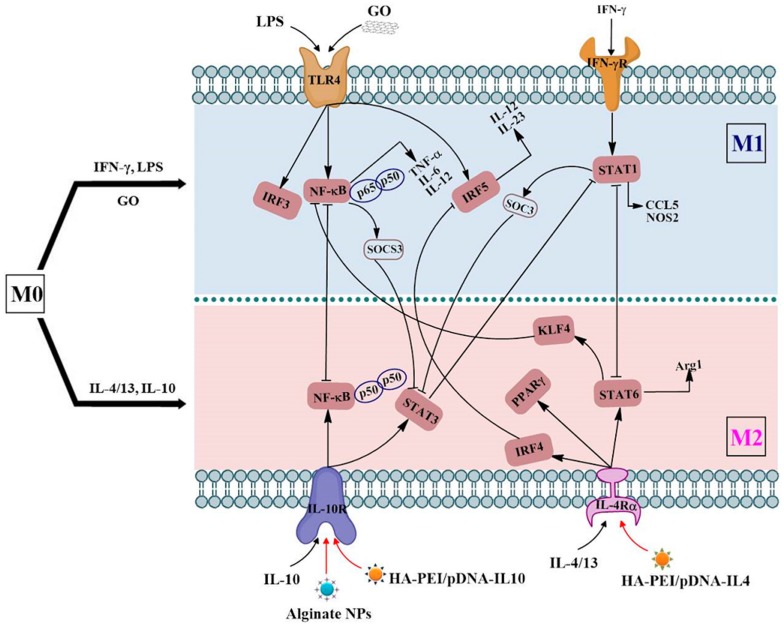
Signaling pathways of macrophage polarization. IRF, STAT, NF-κB, and suppressor of cytokine signaling (SOCS) are the major pathways of macrophage polarization. Krüppel-like factor 4 (KLF-4) is the downstream protein of STAT6. GO (graphene oxide) could induce macrophage polarization towards M1 phenotype [45]. Plasmid DNA expressing IL-10 or IL-4 encapsulated hyaluronic acid-poly (ethyleneimine) NPs (HA-PEI/pDNA-IL-10 or HA-PEI/pDNA-IL-4 NPs) [46] and tuftsin-modified alginate NPs containing murine cytokine IL-10 plasmid DNA [34] modulated macrophage reprogramming from M1 toward M2 (**red arrow**).

Studies have revealed that different receptors on the macrophage handle different signals from the microenvironment (Figure 1), which initiates macrophage polarization [40]. Studies have demonstrated that TLR4 and IFN-γ receptors on the macrophage can interact with the M1 activation signal, while IL-10R and IL-4R respond to the M2 activation signal [41]. Signaling pathways involved in macrophage polarization have been investigated (Figure 1). The major signals associated with M1 macrophage polarization are signal transducers and activators of transcription 1 (STAT1) and nuclear factor κB (NF-κB) [42]. STAT3, STAT6 or peroxisome proliferator-activated receptor-γ (PPAR-γ) activation typically results in M2 macrophage polarization.

Different signals from the microenvironment are handled by different receptors on the macrophage to trigger various polarization pathways (Figure 1). For example, LPS stimulates TLR4 and then activates NF-κB and interferon regulatory factor 3 (IRF3), thus promoting the secretion of pro-inflammatory cytokines, such as IL-6 and TNF-α and metabolic enzyme iNOS, and induce M1 polarization [43]. After interacting with IFN-γR, IFN-γ activates STAT1 and its phosphorylation. After activating the IL-10R receptor, IL-10 subsequently induces STAT3 and p50 NF-κB homodimer and M2 polarization. Through stimulus of IL-4Ra, IL-4, and IL-13 mainly activate STAT6 and cause the expression of M2 phenotype markers, such as Arg-1 [44].

## 3. NPs Modulate Macrophage Polarization and Reprogramming

Studies have demonstrated that different NPs can induce M0 macrophage polarization toward various phenotypes and modulate macrophage reprogramming (Table 1 and Figure 1).

### 3.1. NPs Induce M0 Macrophage toward M1 Polarization

M1 macrophages exhibit potent microbicidal and tumoricidal activity. NPs can induce inflammation in the animal models via M1 polarization [45]. Available data demonstrated that the physicochemical properties of NPs, such as chemical composition [21], size [47], and surface coatings [46], can differentially regulate M1 polarization (Table 1).

#### 3.1.1. Chemical Composition

Metal NPs have a variety of potential applications in the field of electronics, chemistry, energy, and medicine [48]. The pro-inflammatory effects of Ag NPs and Au NPs were compared. Ag NPs (3.08, 5.75, and 24.85 nm) and Au NPs (2.81, 5.52, and 38.05 nm) are similar in size, but the expression levels of pro-inflammatory cytokines IL-1, IL-6, and TNF-α were higher in all sizes of Au NP-treated J774.A1 macrophages than Ag NP-treated groups [47]. This result indicated that Au NPs have a greater effect on inducing M1 macrophage polarization than Ag NPs with the same size. The reason underlying the differential degree of macrophage polarization induced by Au NPs and Ag NPs was also explored [47]. Ag NPs enter the macrophages via pinocytosis, whereas Au NPs can enter cells via receptor-mediated endocytosis and via pinocytosis. The different methods of cellular uptake between these two NPs might contribute to their different ability to induce macrophage polarization [47].

However, others have reported that Ag NPs (15 and 40 nm) exhibited higher propensities in inducing M1 macrophage polarization than Au NPs (20 and 40 nm), indicated by the induction of TNF-α and IL-6 in RAW264.7 macrophages [49]. In this study, Ag NPs elicited a significant increase of NF-κB while Au NPs showed no activation of NF-κB and the inflammatory response induced by Ag NPs was similar to that induced by LPS. In several other studies, Au NPs were also not observed to induce the expression of M1 related markers [50,51,52]. According to available literature, different pro-inflammatory effects of Au NPs in macrophages were related with the length of exposure time. As exposure time extends, the pro-inflammatory response induced by Au NPs decreased. Studies demonstrated that inflammatory response was activated by Au NPs after 3 and 6 h treatment [47] but the expression levels of all pro-inflammatory related markers went down after 24 h treatment [49,50,51,52]. The down-regulation of these pro-inflammatory genes might be induced by the proliferation of macrophages after 24 h [47]. In addition, Co NPs (50–200 nm) were reported to direct primary macrophages toward an inflammatory M1 phenotype by reducing anti-inflammatory IL-1Rα and inducing inflammatory TNF-α secretion in both primary macrophages and LPS-polarized macrophages [21].

Aside from metal NPs, metal oxide NPs are also the commonly used NPs. ZnO, TiO_2_, and Ag NPs are the three major NPs applied in consumer products [29]. Studies have suggested that the pro-inflammatory effect of ultralow concentrations of Ag, TiO_2_, and ZnO NPs in RAW264.7 macrophages were dependent on their doses. As the dose of these NPs increased from 10^−7^ to 10^−3^ μg/mL, the levels of cytokine IL-6 and IL-1β were up-regulated. At the dose of 10^−3^ µg/mL, the highest pro-inflammation response was observed in AgNPs group (IL-6 and IL-1β were 2.0-fold increase relative to the untreated control), whereas the lowest level were observed in ZnO NPs group (IL-6 and IL-1β were 1.4-fold and 1.2-fold, respectively) [29]. Available studies suggested that pro-inflammatory effects of these NPs may be mediated by the activation of NF-κB and subsequent pro-inflammatory gene expression [29,53].

Iron oxide NPs have been widely used as contrast agents and drug carriers in preclinical and clinical settings [54,55]. Recent study revealed that ferumoxytol NPs could significantly inhibit tumor growth by inducing M1 macrophage polarization [12]. In vitro, ferumoxytol NPs increased mRNA associated with pro-inflammatory Th1-type responses in macrophages, which resulted in the increased caspase-3 activity in adenocarcinoma cells co-incubated with ferumoxytol NPs and macrophages. In vivo, ferumoxytol NPs significantly inhibited growth of subcutaneous adenocarcinomas and prevented hepatic metastasis in mice. Further study showed that the increased presence of pro-inflammatory M1 macrophages in the tumor tissues contributed to the observed tumor growth inhibition. These results suggested that ferumoxytol NPs could potentiate macrophage-modulating cancer immunotherapy [12].

The production of SiO_2_ NPs ranks first among ceramic NPs [21]. SiO_2_ NPs (15 nm, 400 μg/10^6^ cells) were observed to stimulate human macrophage (PMA-differentiated myelomonocytic U-937 cells) polarization toward a pro-inflammatory M1 phenotype, as evidenced by highly increased activity of inflammatory cytokines IL-1β (130-fold relative to the untreated control) and TNF-α (about 30-fold relative to the untreated control) [21]. However, other ceramic NPs, such as titanium oxide (TiO_2_) NPs (70 nm) and the more inert zirconium oxide (ZrO_2_) NPs (5–30 nm), did not induce an inflammatory phenotype in primary macrophages. Overall, ceramic NPs of different chemical compositions have distinct effects on macrophage polarization. Compared to inert TiO_2_ and ZrO_2_ NPs, SiO_2_ NPs could dramatically polarize primary macrophage toward the M1 phenotype. The discrepancy among these NPs might result from the up-regulated expression of TLR4 co-receptors CD14 by SiO_2_ NPs, which make macrophages recognize SiO_2_ NPs as LPS [21].

Taken together, the chemical composition of NPs is an important factor that affects the polarization state of macrophages. However, different cell models and doses of NPs were used, so we still lack key databases and face the challenge of comparing various NPs.

#### 3.1.2. Size

Graphene oxide (GO) NPs induced macrophages to polarize to the M1 subtype in a size-dependent manner [45]. The largest GO nanosheets (750 to 1300 nm) secreted the highest levels of pro-inflammatory cytokines TNF-α, IL-6 and IL-1β in both human THP-1 cells and J774.A1 cells, compared to the intermediate (350 to 750 nm) nanosheets. The lowest levels of pro-inflammatory cytokines were observed in the smallest GO nanosheets (50 to 350 nm)-treated groups. Accordingly, the largest GO induced the highest iNOS mRNA (31-fold relative to the untreated control) expression. Further studies have shown that GO size-dependent macrophage polarization was not associated with the quality of cellular uptake of GO [45]. Although the smallest GO had the greatest cellular uptake, it could not activate the TLR4 signaling and NF-κB pathways to induce macrophage polarization. In contrast, the largest GO exhibited less cellular uptake but tended to adsorb to the plasma membrane and generated greater macrophage activation.

However, many other NPs with smaller size were reported to have a more potent effect on inducing M1 polarization than their larger counterparts. For example, the small Au NPs (2.81 nm) were more potent in the induction of J774.A1 macrophages into the M1 subtype than the medium (5.52 nm) and large NPs (38.05 nm), as determined by a higher expression of IL-1, IL-6, and TNF-α [47]. The cytotoxicity of Au NPs decreases as its size increases, which might be a reason for its size-dependent macrophage polarization. For most metallic NPs, the degree of induced macrophage polarization toward the M1 subtype was related to the size of the NPs [49]. As the Ag, Al, and Au NP size decreases, there is a greater effect on M1 macrophage polarization, as indicated by the release of TNF-α and IL-6 in RAW264.7 macrophages [49].

In summary, size plays a determining factor in driving macrophage polarization. However, whether the smaller or larger NPs are more likely to induce macrophage polarization is not consistent among the available reports.

#### 3.1.3. Surface Chemical Modification

Due to their excellent biocompatibility, Au nanorods (NRs) modified with peptides can be used to deliver drugs [27]. Au NRs with different surface modifications of bioactive peptides were observed to induce macrophages toward contrasting polarization states. Bioactive peptides, arginine-glycine-aspartic acid (RGD) or glycine-leucine-phenylalanine (GLF), were attached to the Au NRs by the attachment of the surface layer, polyethylene glycol (PEG) [23]. GLF NRs have a pro-inflammatory effect, unlike RGD NRs which induce M2 macrophage polarization, by directing isolated hepatic macrophages toward the M1 subtype, as evidenced by the up-regulated expression of TNF-α and the low levels of Arg1, IL-4, and Retnla [23]. However, the underlying mechanism of different polarization states induced by different surface modification of Au NRs was not clear.

Taken together, the mechanisms of NPs induced M1 polarization are still obscure based on the recent extremely limited studies. The exact clues are the stimulation of TLR4 and activated NF-κB pathway by NPs skew macrophage toward M1 phenotype. The diverse contact with macrophages and enter ways might contribute to distinct degrees on macrophage polarization.

### 3.2. NPs Drive Primary Macrophage Polarization toward the M2 Phenotype

Compared to M1 macrophage polarization, few reports on NP-induced M2 macrophage polarization are available (Table 1).

M2 macrophages are the key effector cells in the early stages of tissue healing. Polarizing macrophages toward M2 phenotypes is essential for osteogenesis of implanted biomaterial [56]. TiO_2_ NPs are of significant concern in the field of implants because titanium metal resists corrosion from the body. The expression of M2 subtype markers (Arg1, MR, and CD163) was markedly increased on the surface of bioactive ion Ca and Sr-modified nanostructured Ti implants [33]. Further studies showed that surface bioactive ion modification on the nanotopography of Ti implants plays an important role in inducing M2 macrophage polarization by modulating the shape and spread of adherent macrophages. Modulating the M2 polarization by surface nanotopography and chemistry of nanostructured Ti implants provides insight into the basis for the rational design of implants, which orchestrate the early wound healing process and osteogenesis [33].

In addition, Au NRs surface functionalized with RGD were observed to dramatically suppress the expression of M1 markers, such as Arg1 and TNF-α, while increasing the expression of M2 markers, including IL-4 and Retnla, in hepatic macrophages isolated from mouse livers, indicating that RGD rods polarized hepatic macrophages toward the anti-inflammatory M2 macrophage phenotype [23].

### 3.3. NPs Stimulated Macrophage Reprogramming

Studies have suggested that NPs could cause macrophage reprogramming (Table 1). Considering the significance of macrophage reprogramming in modulating the balance of the immune system and the process of related diseases, such as cancer and chronic inflammation, NPs might act as tools in treating such diseases.

#### 3.3.1. The Switch from the M2 Phenotype to M1 Phenotype

Super paramagnetic iron-oxide nanoparticle (SPIONs) could shift macrophages from the M2 phenotype to the M1 phenotype, which is characterized by up-regulated CD86 and TNF-α levels. Further study showed that iron in SPIONs contributed to the phenotypic shift in THP1-derived M2 macrophages toward the M1 phenotype [22]. SPIONs might affect macrophage phenotype by changing the cellular iron concentration [22].

Glycocalyx-mimicking NPs (glycol-NPs) were observed to reverse the M2 phenotype [37]. Glycol-NPs were self-assembled by three sugars, galactopyranoside (Gal), mannopyranoside (Man) and fucopyranoside (Fuc). Glycol-NPs skew the mouse peritoneal macrophage-derived M2 phenotype into an M1 phenotype, indicated by the up-regulated expression of CD86 and IL-12, and the down-regulated expression of CD206, CD23, and IL-10 in vitro. Other studies showed that Fuc plays important roles in glycol-NP-induced M2 phenotypic shift. In contrast to Man and Gal, Fuc caused high expression of CD86 by interacting with specific mannose receptors (MR, CD206) on macrophages. In vivo, peritoneal macrophages from glycol-NP injected mice were also observed to secrete high levels of pro-inflammatory monocyte chemotactic protein 1 (MCP-1) and TNF-α, proof of the polarization switch from M2 to M1 [37]. Further study suggested these glycol-NPs could specifically interact with the receptors expressed on M2 and the receptor-mediated uptake process might induce the phenotypic shift [37].

Polystyrene NPs with surface carboxyl-(PS-COOH) and amino-(PS-NH_2_) groups could strongly skew the M2 macrophage polarization without affecting M1 markers [24]. Furthermore, both PS-COOH and PS-NH_2_ contributed to reducing the expression of M2 surface receptors, CD200R and CD163, along with reducing the secretion of cytokine IL-10, which was essential for the identification of the M2 phenotype. Although these surface functionalized NPs could inhibit M2 polarization, the underlying mechanism is poorly understood. As CD200R and CD163 are known for restraining the inflammatory response, these NPs might be exploited as an anti-inflammation therapy [24].

#### 3.3.2. The Switch from the M1 Phenotype to M2 Phenotype

Plasmid DNA (expressing IL-4 or IL-10)-encapsulated hyaluronic acid-poly (ethyleneimine) NPs (HA-PEI/pDNA) with an average size of 186 nm could modulate the reprogramming of macrophages from the M1 to M2 subtype, confirmed by increased Arg/iNOS levels, higher expression of CD206, and down-regulation of the M1 marker CD86 in J774A.1 macrophages. In vivo models of C57BL/6 mice intraperitoneally injected with LPS and IFN-γ presented notably down-regulated expression of iNOS (from 255-fold to about five-fold relative to the LPS-treated control) level and up-regulated expression of CD163 level following treatment with HA-PEI/pDNA NPs [46]. Further study suggested that targeting CD44-overexpressed peritoneal macrophages by HA and the capacity of PEI for encapsulating nucleic acids made HA-PEI easily internalized by the macrophage. The phenotypic switch induced by HA-PEI/pDNA NPs was related to IL-4 or IL-10 mediated M2 polarization [46].

Another microRNA-223 (miR-223) duplex- and miR-223-expressing plasmid DNA-encapsulated HA-PEI NPs (HA-PEI/miR-223 NPs) also skewed macrophage functional polarity from M1 toward M2, evidenced by the significant decrease in iNOS and increase in Arg-1 levels [57]. In vivo models of LPS-stimulated peritoneal macrophages decreased inflammatory cytokine levels of TNF-α, IL-1β, and IL-6 in HA-PEI/miR-223 NP-treated groups and indicated that these NPs induced the macrophage phenotypic shift from M1 toward the M2 phenotype. miR-223 is a critical regulator of macrophage polarization, which suppresses classic pro-inflammatory pathways and enhances the anti-inflammatory responses [58]. miR-223-encapsulated HA-PEI NPs could induce macrophage phenotypic change toward the anti-inflammatory phenotype and subsequently reducing inflammation, suggesting its potential for the treatment of inflammatory diseases [57].

Tuftsin-modified alginate NPs containing murine cytokine IL-10 plasmid DNA, compared to unmodified NPs, switched the pro-inflammatory M1 subtype to the anti-inflammatory M2 state, indicated by the alleviation in levels of pro-inflammatory cytokines IL-6 (from five-fold to 0.5-fold relative to the LPS-treated control), IL-1β (from 30-fold to four-fold relative to the LPS-treated control) and TNF-α (from 27-fold to 3.9-fold relative to the LPS-treated control), and promotion of the production of the anti-inflammatory cytokine IL-10. This reprogramming provides a solid basis for rheumatoid arthritis treatment [34].

Taken together, the surface functionalized NPs could shift macrophages from pro-inflammatory M1 toward anti-inflammatory M2 phenotype through delivering anti-inflammatory related cytokines miRNA or plasmid DNA, which provides the platform for alleviating systemic inflammatory diseases. The underlying mechanisms are that the delivered substances activate M2 polarization just like the stimulus, such as IL-4 or IL-10 [58].

## 4. Conclusions

Available data have demonstrated that macrophage polarization and reprogramming could be differentially modulated by NPs with different physicochemical properties including chemical composition, size and surface modification. However, a clear generalized conclusion across NPs on how physicochemical properties affect the polarization and reprogramming of macrophage could not be drawn based on the very limited literature. Furthermore, the available studies are still lack of detailed knowledge about the underlying mechanisms. Further study in these fields is urgently needed.

Alterations in the balance of M1/M2 macrophage polarization correlate with the development and progression of a number of diseases. Accompanied with further research, rules of how to utilize the physicochemical properties to control the polarization state of macrophage will be mastered. NPs designed for optimal modulation of macrophage polarization and reprogramming may be good options for treating inflammatory diseases, biomaterials implantation, and tumor immunotherapy.

## Figures and Tables

**Table 1:** NPs induce macrophage polarization and reprogramming.

NPs	Shape	Zeta Potential (mV)	Hydrodynamic Diameter (nm)	Core Size (nm)	Surface Modification	Initial PhenoType	Polarized Phenotype	Model	Cell Line	Marker	Ref.
Ag	-	−11.30 ± 1.01 in medium	168.57 ± 2.47 in medium	35.53 ± 14.92	-	M0	M1	in vitro	RAW264.7	IL-6 , IL-1β↑	[27]
	sphere	13.69 ± 0.25 in H_2_O	-	3.08 ± 1.16					J774.A1	IL-1, IL-6, TNF-α↑	[44]
		15.43 ± 2.72 in H_2_O		5.75 ± 1.12							
		5.35 ± 1.26 in H_2_O		24.85 ± 6.06							
		−39.5 ± 3.5 in H_2_O		15 ± 3					RAW 264.7	TNF-α, IL-6↑	[47]
		−34.5 ± 3.7 in H_2_O		40 ± 4							
Au	sphere	−56.64 ± 1.84 in H_2_O	-	2.81 ± 0.84	-	M0	M1	in vitro	J774.A1	IL-1, IL-6, TNF-α↑	[44]
		−60.85 ± 2.88 in H_2_O		5.52 ± 0.95							
		−78.81 ± 1.97 in H_2_O		38.05 ± 11.88							
Co	-	-	-	50–200	-	M0	M1	in vitro	U-937	TNF-α↑	[19]
ZnO	-	−8.62 ± 0.26 in medium	89.92 ± 1.58 in medium	31.89 ± 12.63	-	M0	M1	in vitro	RAW264.7	IL-6 , IL-1β↑	[27]
TiO_2_	-	−11.20 ± 1.11 in medium	191.57 ± 1.52 in medium	30.70 ± 9.18	-	M0	M1	in vitro	RAW264.7	IL-6 , IL-1β↑	[27]
SiO_2_	-	-	-	15	-	M0	M1	in vitro	U-937	IL-1β, TNF-α↑	[21]
Ferumoxytol	-	-	-	15 nm	Carboxymet-hyldextran	M0	M1	in vitro	RAW264.7	CD86, TNF-α↑	[12]
								in vivo	Liver and lung macrophages	CD80↑ CD206↓ (liver) CD206↓ (lung)	
GO	sheet	−31.88 ± 2.42 in H2O	-	50–350	-	M0	M1	in vitro	J774.A1 and THP-1	TNF-α, IL-6, iNOS↑ (J774.A1) & IL-1β, TNF-α↑ (THP-1)	[45]
		−30.42 ± 2.17 in H_2_O		350–750							
		−28.72 ± 2.36 in H_2_O		750–1300							
Au	rod	-	-	15 × 50	GLF	M0	M1	isolated from mouse liver	mouse hepatic macrophage	Arg1, Retnla, IL-4↓, TNF-α↑	[23]
					RGD	M0	M2			Arg1, Retnla, IL-4↑, TNF-α↓	
Ti	-	-	-	30–50	Ca and Sr	M0	M2	in vitro	J774.A1	Arg1, MR, CD163↑	[33]
Glyco-NP	-	-	36 in PBS	-	Gal	M2	M1	isolated from mouse peritoneal cavity	mouse peritoneal macrophage	CD86, IL-12↑, CD206, CD23, IL-10↓, TNF-α↑	[37]
			34 in PBS		Man						
			34 in PBS		Fuc						
SPIONs	-	−8.02 in 0.9% NaCl	-	60.32	-	M2	M1	in vitro	THP1 derived M2 macrophage	CD86, TNF-a↑	[22]
HA-PEI NPs	-	-	185.9 in PBS	80–120	plasmid DNA IL-10	M1	M2	in vitro & isolated from mouse peritoneal cavity	J774.A1 & mouse peritoneal macrophage	Arg, CD163, IL-10↑, iNOS, CD80↓ (J774.A1) CD163, IL-10↑ TNF-α, IL-1β, iNOS↓ (peritoneal macrophage)	[47]
					plasmid DNA IL-4					Arg, CD206, CD163, IL-10↑, iNOS, CD80↓ (J774.A1) CD163, IL-10↑ iNOS↓ (peritoneal macrophage)	
		−14.7 in PBS	~200 in PBS	~200	miR-223 plasmid DNA encapsulated					IL-1β, TNF-α, IL-6, iNOS↓, Arg↑in two cell lines	[57]
Alginate NPs	sphere	15.8 ± 3.7	299.7 ± 2.2	180–250	mIL-10 plasmid DNA	M1	M2	in vitro	synovial macrophage	IL-6, IL-1β, TNF-α↓	[34]

Upward arrow (↑) and downward arrow (↓) represented the increased or decreased expression of markers respectively.
